# Potential Cost-Savings From the Use of the Biosimilars in Slovakia

**DOI:** 10.3389/fpubh.2020.00431

**Published:** 2020-08-21

**Authors:** Tomas Tesar, Peter Golias, Zuzana Kobliskova, Martin Wawruch, Paweł Kawalec, András Inotai

**Affiliations:** ^1^Department of Organisation and Management in Pharmacy, Faculty of Pharmacy, Comenius University in Bratislava, Bratislava, Slovakia; ^2^Institute for Economic and Social Reforms, Bratislava, Slovakia; ^3^Faculty of Medicine, Institute of Pharmacology and Clinical Pharmacology, Comenius University in Bratislava, Bratislava, Slovakia; ^4^Faculty of Health Sciences, Institute of Public Health, Jagiellonian University Medical College, Krakow, Poland; ^5^Syreon Research Institute, Budapest, Hungary; ^6^Center of Health Technology Assessment, Semmelweis University, Budapest, Hungary

**Keywords:** biomedical, decision making, insurance, health, reimbursement, health policy, savings, Slovakia

## Abstract

**Objectives:** To analyse the market shares of biosimilars in Slovakia and to calculate the potential cost-savings from the use of biosimilars in Slovakia based on two different data sources.

**Methods:** National reimbursement lists from the Czech Republic, Hungary, Poland and Slovakia were used for analyzing the availability of biosimilars with public funding. In addition, the reimbursement dossiers of biosimilars, the justifications of reimbursement decisions by the Slovak Ministry of Health, and final reimbursement decrees, which are published on the webpage of the Slovak Ministry of Health, were utilized for this study. Reimbursement decisions regarding biosimilars by the Slovak Ministry of Health from 2006 to August 2019 were considered and the detailed utilization of biosimilars in 2018 was analyzed based on data from the State Institute for Drug Control. The study was validated based on data from the Slovak National Health Information Center.

**Results:** Fifty four biosimilars were approved by the European Medicines Agency (EMA) in August 2019. Of the total group of licensed biosimilars on the market, 29 biosimilars (54%) were available in the Czech Republic, 28 biosimilars (52%) were available in Poland, and 27 biosimilars (50%) were available in Hungary and 24 biosimilars (44%) were available in Slovakia. Our analysis, based on the data provided by distributors of medicinal products to the State Institute for Drug Control, revealed that the health fund in Slovakia could have saved 35 to 50 million euros per year if biosimilars with marketing authorisations had been available on the Slovak market. The calculations assumed a 25–35% price decrease against the original biological medical products, and that there would be no increase in the utilization of biosimilars in Slovakia.

**Conclusions:** To achieve significant improvement in patient access to biosimilars in Slovakia, a top-down approach establishing targets and quotas for the procurement of biosimilars should be applied.

## Introduction

According to the European Medicines Agency (EMA), biological medicines consist of active components from biological sources such as living cells or organisms and most biologicals are produced by biotechnology, usually using sophisticated cell systems and recombinant DNA technology (1). Marketing authorization is granted to medicinal products when studies on their quality, safety and efficacy convincingly demonstrate that the medicine's benefits outweigh the risks (2). Geynisman (3) concluded that the entry of biological medicinal products has substantially changed the treatment of serious and chronic conditions such as diabetes, autoimmune diseases and cancer.

A biosimilar medicine is greatly similar to another biological medicine, known as a “reference medicine” (4). Marketing authorization holders can market approved biosimilars after the patent expiration of reference medicine. Rigorous controls are always in place during manufacturing to ensure that minor differences among the biosimilars and their reference medicines do not affect safety and efficacy and that the differences are not clinically meaningful (1).

Kurki (5) concluded that most of the best-selling reference biologicals are or will soon be facing competition from biosimilars in the European Union.

However, Markus (6) emphasized that despite the application of rigorous norms, a potential doubt about biosimilar medicines was the extrapolation of clinical data required for the registration of all indications of the reference medicine. According to Strand (7) a systematic literature review found that immunogenicity of biosimilar medicines differs among active compounds, suggesting that the immunogenicity of anti-drug antibodies should be an important consideration in the therapy decision-making process such as switching. It could be seen that the utilization of biosimilar medicinal products, especially for patients on original biological maintenance treatment, was not an evident preference for many physicians (8–10).

On the other hand, other systematic reviews found that switching patients from chronic biologic therapy to a biosimilar alternative was not associated with an increased risk of adverse reactions or loss of efficacy (5, 11).

Cook (12) stressed that concerns of clinicians related to safety, efficacy and cost, will need to be addressed before they are prescribing biosimilars.

Results from a recently published systematic review demonstrate that physicians in Europe and the U.S. do not primarily support the use of biosimilar medicinal products as safe and effective pharmacotherapies for patients already getting reference biological treatment (13).

The outcomes of a study performed by the European Society for Medical Oncology (ESMO) on biosimilar understanding among oncologists emphasized the need for education and worldwide alignment (14).

However, physicians in the European Crohn's and Colitis Organization (ECCO) have stated that switching from the reference biological medicinal product to a biosimilar for patients with inflammatory bowel disease is acceptable (15).

In Norway, physicians envisaged first-line use of biosimilar medicines for biologic naïve patients with inflammatory bowel disease (16) and in Denmark, a similar approach has been supported by the national council for expensive hospital medicines in rheumatology and gastroenterology (17).

According to Moorkens (18) Swedish specialists prescribing TNF α inhibitors are in compliance with the recommendation of the Swedish Medical Products Agency, which considers pharmacotherapy with a biosimilar medicinal product uncontroversial in treatment-naïve patients and believes no barriers exist to switch stable, well-informed patients from the reference biological medicinal product to the biosimilar; the recommendation also stated that more data on multiple switching are needed.

The introduction of biosimilars may create competition for biological medicinal products, possibly resulting in reduced prices, and altered market dynamics in disease areas (18).

Because of its high development costs, the biosimilar medicinal product can only be introduced on the pharmaceutical market with a limited discount compared to the reference medicine (19). Although they are usually priced at a discount of only 10–35% (20) absolute cost savings could still be significant because of the high prices and volumes of the reference medicines (21).

Hidden volume constraints to biologicals implemented by payers to facilitate the financial sustainability of health care systems may increase the European inequity in patient access to treatment (22).

Kaló (22) argued that there is a substantial contrast in the utilization of biological medicinal products in the European Union, as biologicals at Western European price levels are usually not cost-effective in Central and Eastern European countries.

Kaló (23) pointed out that the launch price of high-cost biologic medicines is set according to the highest acceptable price by payers in large and high-income countries with the greatest market potential. These prices are frequently not acceptable in lower income countries (24).

Policymakers in healthcare systems should take action to increase the use of biosimilar medicines, partly by handling concerns related to their efficacy and safety raised by different stakeholders (19, 25).

The Slovak Ministry of Health (26, 27) stated that the ex-factory prices of biosimilars approved for the Slovak reimbursement list may not exceed the average of the three lowest prices of the same biosimilars available on pharmaceutical markets across the European Union.

The situation with availability of biosimilars was compared among V4 countries. The Visegrád Group, or V4, is considered as a cultural and political alliance of the Czech Republic, Hungary, Poland, and Slovak Republic.

Kuenzel (28) highlighted that Slovakia is among the EU Member States with the greatest potential to improve health outcomes without raising costs.

The implementation of policy practices that maximize the social benefits of biosimilars for patients in Slovakia should be approved. This paper is aimed at examining the lost opportunities for savings due to the restrained availability of biosimilars in the Slovak healthcare system.

## Materials and Methods

The publicly available EMA website (29) was searched to obtain the list of biosimilars for which the EMA granted marketing authorization.

The analysis covered the following elements: (a) the reimbursement status of biosimilars in the Czech Republic, Hungary, Poland, and Slovakia, (b) a comparative study of the market shares of biosimilars in Slovakia and (c) a calculation of potential cost-savings that could be achieved through the use of biosimilars in Slovakia based on two different data sources.

The reimbursement status of biosimilars in the Visegrád Group was analyzed based on the national reimbursement lists of medicinal products, which was valid in the countries for August 2019 (30–33). If a biosimilar was available in the abovementioned reimbursement lists, we considered it available for patients in a particular country.

Coverage decisions regarding biosimilar medicinal products made by the Slovak Ministry of Health from 2006 to August 2019 were studied more deeply and the consumption of biosimilars in 2018 was analyzed.

We assumed a 25–35% price decrease (34) against that of reference products for the calculation of potential cost-savings from the use of the biosimilars in Slovakia. The Slovak Ministry of Health (27) required that the first biosimilar entering the reimbursement list had to provide a 30% initial price decrease compared to the price of the reference drug for 2018. The second biosimilar entering the reimbursement list had to offer an additional 5% price reduction compared to that of the first biosimilar, and the third biosimilar had to offer an additional 5% price reduction compared to that of the second biosimilar. In addition, it was assumed that there would be no increase in the utilization of the analyzed biosimilars in Slovakia.

Pursuant to updated legislation for 2019 (27) the first biosimilar entering the reimbursement list has to provide a 25% initial price decrease compared to that of the reference medicine. The price reduction requirements for the second and third biosimilars in 2019 remained the same as for 2018.

Based on the 5th level of the Anatomical Therapeutic Chemical Classification System (ATC5), financial expenditures of the Slovak health insurance funds for reference medicines with biosimilar alternatives approved by the EMA which were not present in the Slovak reimbursement list in 2018 were analyzed and the potential cost-savings expected from the use of the biosimilars in the case of their availability on the Slovak reimbursement list was calculated. We estimated saving potential based on a 25–35% decrease in financial expenditures of Slovak health insurance funds for the ATC5 of reference medicines with those EMA-approved biosimilar alternatives, which were not on the Slovak reimbursement list in 2018. We estimated no additional saving potential for reference biological medicines with EMA-approved biosimilar alternatives, which were on the Slovak reimbursement list in 2018 because in those ATC5, cost-savings had already been achieved. Additionally, we calculated no potential cost-savings for the subcutaneous formulation of Trastuzumab and Rituximab, without biosimilar alternatives approved by the EMA. However, we incorporated potential cost-savings for the intravenous formulation of Trastuzumab and Rituximab with biosimilar alternatives already approved by the EMA.

Data from distributors of medicinal products, which are required to deliver sales data to the State Institute for Drug Control, were used for this study (35). This database is not publicly available and there is a fee for its access. The State Institute for Drug Control is the state authority in the field of human pharmacy and drug precursors (36). A confirmatory study was also performed to validate the results. Reimbursement data from payers, which are required to provide data to the National Health Information Center, were used for validation purposes. This database is publicly available and access to it is free of charge. The National Health Information Center performs tasks in the area of health statistics and the provision of library and information services in the field of medical sciences and health services (37).

## Results

Table 1 shows information on the availability of biosimilars with public funding in the Czech Republic, Poland, Hungary and Slovakia in August 2019. Of the 54 biosimilars approved in August 2019 by the EMA, 29 biosimilars (54%) were available in the Czech Republic, 28 biosimilars (52%) were available in Poland, and 27 biosimilars (50%) were available in Hungary, 24 biosimilars (44%) were available in Slovakia.

**Table 1:** Availability of biosimilars in Slovakia in August 2019.

**Year of EMA approval**	**Active substance (medicinal product)**	**Slovakia**	**Czech Republic**	**Poland**	**Hungary**
2017	Adalimumab (Amgevita)	Available	Available	Available	Available
2018	Adalimumab (Hefiya)				
2018	Adalimumab (Hulio)	Available	Available		Available
2018	Adalimumab (Hyrimoz)	Available	Available	Available	Available
2017	Adalimumab (Imraldi)		Available	Available	
2018	Adalimumab (Halimatoz)				
2019	Adalimumab (Kromeya)				
2019	Adalimumab (Idacio)		Available		Available
2018	Bevacizumab (Mvasi)				
2019	Bevacizumab (Zirabev)				
2016	Enoxaparin sodium (Inhixa)				
2016	Enoxaparin sodium (Thorinane)				
2018	Epoetin alfa (Abseamed)				
2007	Epoetin alfa (Binocrit)	Available	Available	Available	Available
2007	Epoetin alfa (Epoetin Alfa Hexal)				
2007	Epoetin zeta (Retacrit)				Available
2007	Epoetin zeta (Silapo)				
2016	Etanercept (Benepali)	Available	Available	Available	
2017	Etanercept (Erelzi)			Available	
2014	Filgrastim (Accofil)	Available	Available	Available	Available
2009	Filgrastim (Filgrastim Hexal)				
2013	Filgrastim (Grastofil)	Available		Available	
2010	Filgrastim (Nivestim)		Available	Available	Available
2008	Filgrastim (Ratiograstim)	Available			Available
2008	Filgrastim (Tevagrastim)		Available	Available	
2009	Filgrastim (Zarzio)	Available	Available	Available	Available
2014	Follitropin alfa (Bemfola)	Available	Available	Available	Available
2013	Follitropin alfa (Ovaleap)		Available		Available
2016	Infliximab (Flixabi)		Available	Available	
2013	Infliximab (Inflectra)	Available	Available	Available	Available
2013	Infliximab (Remsima)	Available	Available	Available	Available
2018	Infliximab (Zessly)	Available	Available	Available	Available
2014	Insulin glargine (Abasaglar)	Available	Available	Available	Available
2018	Insulin glargine (Semglee)	Available	Available	Available	
2017	Insulin lispro (Insulin lispro Sanofi)			Available	
2018	Pegfilgrastim (Pelgraz)	Available	Available	Available	Available
2018	Pegfilgrastim (Pelmeg)	Available	Available	Available	Available
2018	Pegfilgrastim (Udenyca)				
2018	Pegfilgrastim (Ziextenzo)	Available	Available	Available	Available
2018	Pegfilgrastim (Fulphila)				
2019	Pegfilgrastim (Grasustek)				
2017	Rituximab (Blitzima)	Available			
2017	Rituximab (Ritemvia)				
2017	Rituximab (Rixathon)	Available	Available		
2017	Rituximab (Riximyo)				
2017	Rituximab (Truxima)		Available		Available
2006	Somatropin (Omnitrope)		Available	Available	Available
2017	Teriparatide (Movymia)				Available
2017	Teriparatide (Terrosa)	Available			Available
2018	Trastutumab (Trazimera)			Available	Available
2018	Trastuzumab (Herzuma)	Available	Available	Available	Available
2018	Trastuzumab (Kanjinti)	Available	Available	Available	Available
2018	Trastuzumab (Ogivri)	Available	Available	Available	
2017	Trastuzumab (Ontruzant)		Available	Available	Available

The overview presented in Table 1 provides a more accurate picture of the market share of selected biosimilar drugs in Slovakia. According to the level of reimbursement for medicines with a given active substance, biosimilar drugs based on the filgrastim molecule (99.93% in 2018) and erythropoietin (75.44%) had the largest market share, followed by insulin glargine (36.29%), infliximab (25.07%), and follitropin alfa (21.11%). Since the end of 2018, the biosimilar medicines containing the active substances rituximab (2.31%) and trastuzumab (0.07%) have also been also marketed. No biosimilar drugs were available for the remaining eight active substances on the market in 2018. By August 2019, adalimumab, pegfilgrastim, etanercept, and teriparatide were added to the categorized biosimilar drug molecules, while bevacizumab, somatotropin, enoxaparin, and insulin lispro molecules remain without a categorized biosimilar drug.

Sales data from wholesalers provided to the State Institute for Drug Control, were used for analyzing the opportunity cost. The results summarized in Table 2 show that 35.44–49.62 million euros per year could have been saved, assuming a 25–35% price against that of original biologic medical products and that there would be no increase in the utilization of biosimilars with better affordability in Slovakia.

**Table 2:** Estimate of financial savings from biosimilars, based on data from the State Institute for Drug Control.

**Active substance**	**Expenditures for ATC5, 2018 (€)**	**Penetration of biosimilars as expenditures for active substances, 2018 (€)**	**Penetration of biosimilars as share of expenditures of active substances (%)/ATC5, 2018**	**Estimate of saving potential based on 25–35% decrease in price of biosimilars compared to original biologicals (mil. €)**
Adalimumab	58,258,594	0	0%	14.56–20.39
Bevacizumab	23,756,125	0	0%	5.94–8.31
Infliximab	15,950,189	3,999,203	25.07%	0
Trastuzumab	13,172,712	9,815	0.07%	3.29–4.61
Rituximab	11,321,961	261,670	2.31%	2.83–3.96
Etanercept	10,504,515	0	0%	2.63–3.68
Erytropoetin	9,272,245	6,994,905	75.44%	0
Somatotropin	9,256,943	0	0%	2.31–3.24
Inzulin glargin	9,136,893	3,316,101	36.29%	0
Enoxaparin	5,983,258	0	0%	1.50–2.09
Pegfilgrastim	5,201,865	0	0%	1.30–1.82
Inzulin lispro	3,388,887	0	0%	0.85–1.19
Filgrastim	2,029,227	2,027,827	99.93%	0
Follitropin alfa	986,869	208,284	21.11%	0
Teriparatid	934,017	0	0%	0.23–0.33
**Total**	**179,154,300**	**16,817,805**	**9.39%**	**35.44–49.62**

Data from payers provided to the National Health Information Center were used for validation purposes. The results summarized in Table 3 show that 26.65–37.32 million euros per year could have been saved assuming a 25–35% price decrease against that of original biologic medical products and that there would be no increase in the utilization of particular biosimilar medicines in Slovakia.

**Table 3:** Estimate of financial savings from biosimilars, based on data from the National Health Information Center.

**Active substance**	**Expenditures for ATC5, 2018 (€)**	**Estimate of potential savings based on 25–35% decrease in price of biosimilars compared to original biologicals (mil. €)**
Adalimumab	35,931,296	8.98–12.58
Bevacizumab	20,416,672	5.10–7.15
Trastuzumab	10,615,228	2.65–3.72
Etanercept	9,609,256	2.40–3.36
Rituximab	9,064,718	2.27–3.17
Somatotropín	8,001,547	2.00–2.80
Pegfilgrastím	4,737,899	1.18–1.66
Enoxaparin	4,268,242	1.07–1.49
Inzulín lispro	3,271,879	0.82–1.15
Teriparatid	700,371	0.18–0.25
**Total**	**106,617,108**	**26.65–37.32**

## Discussion

Inotai (24) argued that the main aim of public healthcare payers is to enhance the allocative efficiency of healthcare spending.

The high launch prices of original medicines, including biologics, may necessitate confidential price discounts, using managed entry agreements or, sub-optimally, the implementation of transparent or hidden access restrictions in order to ensure their financial sustainability (22).

The objective of biosimilar medicine policy, provided that patients have full access to the relevant original biologic products prior to the expiration of their patent, is usually defined as reducing health expenditures without compromising health outcomes (24). Mulcahy (38) demonstrated that a reduction in drug costs can be expected not just from the introduction of biosimilar medicines, but also from the leveraging of competition so as to alter the market dynamics between the biosimilar and reference medicine and their associated prices.

In 2017 and 2018, the EMA authorized biosimilar medicines with the following six active substances with patent expiry: adalimumab bevacizumab, trastuzumab, rituximab, insulin lispro, and teriparatide. Between July 2018 and July 2019, medicines containing the active substances adalimumab, trastuzumab, rituximab, etanercept, and teriparatide were added to the categorization lists in Slovakia. Despite this, of the molecules available in at least one V4 country, biosimilar drugs containing the active substances somatotropin, enoxaparin, and insulin lispro are still absent in Slovakia.

Looking back, over the period up to 2012, four biosimilar drugs were added to the reimbursement list in Slovakia, one of which was later removed at the request of the marketing authorization holder. Two more medicines were added in 2013, one in 2014 and three in 2015. No new biosimilar drugs were added to the reimbursement list in 2016, 2017, or the first half of 2018. These figures presented in Table 1 confirm that Slovakia has an ongoing problem with the availability of biosimilar medicines on the market.

**Figure 1:**
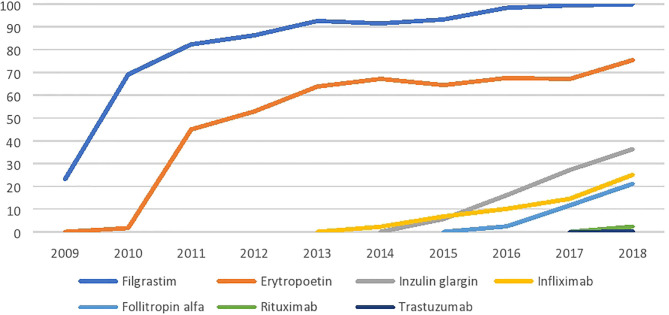
Biosimilar penetration in Slovakia (%).

However, since the second half of 2018 up to May 2019, a fundamental change occurred, as during this period, the Ministry of Health decided to add 15 additional biosimilar drugs to the reimbursement list. The change was partially related to the expiry of the patent protection of several reference medicines, but also to the reduction of the initial compulsory price discount for the first biosimilar product entering the market (i.e., the mandatory price reduction of 30% on the original price was reduced to 25% from 1 January 2019). Longitudinal analyses of biosimilar drug shares by molecule in the Slovak market show that there was a sharp increase in the filgrastim and erythropoietin molecules after their entry to the market in 2009–10, followed by a slowdown in growth and a plateau at high utilization levels. Insulin glargine, infliximab, and follitropin alpha molecules, where biosimilar drugs came to the market later, gradually increased in share. Biosimilar drugs with rituximab and trastuzumab did not enter the market until the end of 2018; therefore, their penetration rates are still low (Figure 1).

Kaló (39) emphasized that the objective of an off-patent drug policy, assuming that patients had full access to the original products prior to their patent expiry, is usually defined as being to reduce health expenditures without compromising health outcomes. Our study has provided an estimate of the lost opportunity for savings due to the restricted availability of biosimilar medicines in Slovakia. The estimate of potential savings in Slovakia due to biosimilars with marketing authorisations of those active compounds that are currently in a monopoly position is presented in Table 2 (original analysis, 35–50 million euros per year) and Table 3 (validation, 27–37 million euros per year). Tesar (40) explained that the differences between the data from distributors of medicinal products and payers may be due to the parallel export of medicines.

Kawalec (2) and Moorkens (41) investigated the price regulation of biosimilar drugs in various European countries. Tesar (40) found that in 2018 the first biosimilar entering the Slovak market had to have a 30% initial price reduction against the price of the original biologic medicine; the second biosimilar had to be launched with an additional 5% reduction against the first biosimilar, and the third biosimilar had to have an additional 5% reduction against the second biosimilar. As even the entry of a new package for an already included biosimilar product was considered a new product from the aspect of compulsory price reductions, biosimilar drug manufacturers were motivated to limit re-packaging their products.

In Slovakia, on the other hand, international price referencing significantly reduces drug list prices. The European reference price of a medical product is defined in Slovakia by Act No. 363/2011 Art. 2(f) as “the average of the three lowest prices from among the officially determined prices of the medicinal product in other Member States” (27). Most EU members regulate prices based on referrals. Paradoxically, for countries that are referenced by several other countries, manufacturers are motivated to maintain higher list prices even for less affluent countries, so that list prices do not drop across the European market. This may deter manufacturers from offering greater list price reductions, especially for small countries with limited market potential. Conversely, if a manufacturer agrees to market its medicine at a significantly reduced list price, this could lead to a domino effect in price reduction across all of the countries comparing their list prices with the lowest price countries. To avoid such negative effects, manufacturers keep the list prices high, even in less affluent countries, or delay the launch of the medicine.

It is therefore important for buyers to set appropriate price reduction rules. From an economic point of view, price regulation is needed unless fair competition generates a market price. This applies to monopolistic suppliers, oligopolies, or price cartels. However, if there are several competitors on the market and the authorities reduce the risk of a cartel, such price regulation is not needed. In the case of original reference biologic medicines and the gradual launch of first biosimilars, price regulation (i.e., mandatory initial price reduction compared to the reference medicine) is necessary. However, if several competing medicines are available on the market, price regulation may have a limited impact (i.e., competition generates the market price). In the countries examined by Kawalec (2) and Moorkens (41), the price of biologic medicines administered in hospitals is primarily influenced by tenders of biosimilar medicines in which suppliers compete by providing price discounts to win the tender. In contrast to mandatory initial discounts required by legislation, discounts achieved in tenders are usually not published and, therefore, do not affect and are not affected by international price referencing.

Significant initial mandatory price reductions (e.g., 30% before 2019) in combination with strict conditions for international price referencing may discourage biosimilar drug manufacturers from entering the market in Slovakia. This may extend the monopoly position of some original biopharmaceuticals, reduce price competition, and ultimately limit the cost saving potential of biosimilars in Slovakia.

Countries need to implement a longer-term biosimilar strategy. The following policy practices should be implemented to maximize the social benefits of biosimilars [adapted from Inotai (24)]:

□ Administrative tools and policy measures should be implemented to incentivize the use of more affordable biosimilars;
□ The pricing and reimbursement processes for biosimilars should be expedited to facilitate their prompt market entry;
□ Amendments to clinical guidelines recommending the extended use of biosimilars should be implemented if justified by health benefits, such as providing patients with improved or earlier access to biological therapy;
□ Off-patent biologics (including biosimilars) should be set as the preferred first-line biological therapy for treatment-naive patients; other, still patent-protected biologic medicines with no or limited added benefit should be used only in subsequent treatment lines;
□ After the expiration of a patent, patients should be switched, under medical supervision, from the original biologic medicine to the more affordable biosimilar alternative;
□ There should be no separate reimbursement categories for biosimilars and original biologics with the same active compound or slightly modified formulations (e.g., subcutaneous vs. intravenous forms), unless the modified formulation has significant and proven added benefits to patients or healthcare systems (42);
□ In addition to being informed about scientific evidence on biosimilars, physicians should be guided on how to appropriately educate their patients regarding these medicines; and
□ Information exchange platforms on good practices related to biosimilars between EU Member States should be established.

The current perception and knowledge among physicians in the Slovak Republic regarding biosimilars in comparison with original biologics is unknown. The lack of information in this field represents a limitation of our research, which will be mitigated by the publication of the results of ongoing studies. The first assumption, concerning a 25–35% price decrease against that of reference products, represents one of the limitations of our study, because some higher price decreases could be offered in the case of stronger market competition in particular groups of biosimilars. The second assumption, concerning no increase in the consumption of analyzed biosimilar medicines in Slovakia, represents a limitation of our study, as well. There is a possibility for an increase in the consumption of the analyzed biosimilars in comparison with the reference biological medicines in Slovakia and therefore the potential cost-savings would be lower. On the other hand, the role of biosimilars is not only about cost-saving potential but improving patient access to needed biological pharmacotherapy and ultimately to improve health status of the society.

The timeline can also be considered as a limitation of this analysis. The Slovak Ministry of Health required that the first biosimilar entering the reimbursement list had to offer a 20% initial price reduction in comparison with the price of the reference biological medicine between 2013 and 2018 and there were no requirements for price reductions for the biosimilars added to the Slovak reimbursement list for the same ATC group. From 2018, the first biosimilar entering the reimbursement list had to offer a 30% initial price reduction compared with the price of the reference drug and there are price reduction requirements for the second and the third biosimilars (27). The timeline for analyzing the impact of the Slovak legislation related to biosimilars, which came into force from 2018, is relatively short.

It needs to be stated that the generalization of our results is limited. However, while we discussed the issues of Slovakia, we believe that even other countries with low availability of biosimilars can benefit from this research. The aim of biosimilar policies can be determined differently in countries with significant resource limitations, where access to high-cost biologics is restricted (22, 24, 43).

Despite recent changes in the Slovak legislation, the availability and penetration of biosimilars in the Slovak pharmaceutical market remains limited. This prevents buyers from exploiting the full cost-saving potential of biosimilars. Policies aimed at maximizing the social benefits of biosimilars in Slovakia should be implemented.

## Data Availability Statement

All datasets generated for this study are included in the article/supplementary material.

## Author Contributions

TT, PG, ZK, MW, PK, and AI conceived the conception, design of the study, and contributed in acquisition of data. TT prepared the draft of the manuscript. All authors contributed to the article and approved the submitted version.

## Conflict of Interest

The authors declare that the research was conducted in the absence of any commercial or financial relationships that could be construed as a potential conflict of interest. The reviewer MM declared a past co-authorship with the authors to the handling Editor.
